# Application of the automated haematology analyzer XN-30 for discovery and development of anti-malarial drugs

**DOI:** 10.1186/s12936-019-2642-0

**Published:** 2019-01-14

**Authors:** Takahiro Tougan, Yuji Toya, Kinya Uchihashi, Toshihiro Horii

**Affiliations:** 1 Department of Molecular Protozoology, Research Institute for Microbial Diseases, Osaka University, 3-1 Yamadaoka, Suita, Osaka 565-0871 Japan; 2 Sysmex Corporation, 4-4-4 Takatsukadai Nishiku, Kobe, Hyogo 651-2271 Japan

**Keywords:** XN-30 analyzer, *Plasmodium falciparum*, Erythrocytic stage, Anti-malarial drug, In vitro drug screening, Medicines for Malaria Venture, Pathogen Box

## Abstract

**Background:**

The erythrocytic stage of *Plasmodium falciparum* parasites in humans is clinically important, as the parasites at this growth stage causes malarial symptoms. Most of the currently available anti-malarial drugs target this stage, but the emergence and spread of parasites resistant to anti-malarial drugs are a major challenge to global eradication efforts; therefore, the development of novel medicines is urgently required. In this study, the in vitro anti-malarial activity of five current anti-malarial drugs (artemisinin, atovaquone, chloroquine, mefloquine, and pyrimethamine) and 400 compounds from the Pathogen Box provided by the Medicines for Malaria Venture on *P. falciparum* parasites was characterized using the XN-30 analyzer. Furthermore, the outcomes obtained using the analyser were classified according to the parasitaemias of total and each developmental stages.

**Results:**

The growth inhibition rate and the half-maximal (50%) inhibitory concentration (IC_50_) of the five current anti-malarial drugs were calculated from the parasitaemia detected using the XN-30 analyzer. Respective strains and drugs presented strongly fitted sigmoidal curves, and the median SD at all tested concentrations was 1.6, suggesting that the variation in values measured with the analyser was acceptably low for the comparison of drug efficacy. Furthermore, the anti-malarial activity of the 400 compounds from the Pathogen Box was tested, and 141 drugs were found to be effective. In addition, the efficacy was classified into 4 types (Type I, parasites were arrested or killed without DNA replication; Type II, parasites were arrested or killed similar to Type I, and the parasitaemia was apparently decreased; Type III, parasites progressed to trophozoite without sufficient DNA replication; and Type IV, parasites were arrested at late trophozoite or schizont after DNA replication).

**Conclusion:**

The current study demonstrates that the XN-30 analyzer objectively, reproducibly, and easily evaluated and characterized the anti-malarial efficacy of various compounds. The results indicate the potential of the XN-30 analyzer as a powerful tool for drug discovery and development in addition to its use as an important diagnostic tool.

**Electronic supplementary material:**

The online version of this article (10.1186/s12936-019-2642-0) contains supplementary material, which is available to authorized users.

## Background

*Plasmodium falciparum* parasites at erythrocytic stage can cause severe malarial symptoms in humans, including fever, anaemia, splenomegaly, and sometimes death [1]. Most of the currently available anti-malarial drugs target this stage [2], but the emergence and spread of anti-malarial drug resistant parasites are a major challenge to global eradication efforts [3]. Therefore, the development of novel medicines is urgently required.

An automated haematology analyser, XN-30 (Sysmex, Kobe, Japan), uses flow cytometry to detect *P. falciparum* in a clinical sample in approximately 1 min [4, 5]. In brief, the XN-30 analyzer aspirates and dilutes blood samples in diluent (CELLPACK DCL). The samples are then treated with a lysis solution (Lysercell M) and the nucleic acids are stained with a dye solution (Fluorocell M). Accordingly, infected red blood cells (iRBCs) are detected using a blue semiconductor 405 nm laser beam, and a sheath flow direct count is used to measure 10 haematological parameters, including the total RBC counts [5]. Further, the XN-30 analyzer has been equipped with an algorithm for in vitro cultured parasites to accurately measure parasitaemia, as well as differentiate and quantitate the developmental stages of parasites [5]. The XN-30 analyzer has also been shown to distinguish iRBCs from white blood cells, polychromatic RBCs, Howell-Jolly body-containing RBCs, and merozoites in a rodent malaria model in vivo. Finally, the XN-30 analyzer has been used to measure parasitaemia after treatment with the anti-malarial drug artemisinin in vivo [6].

In this study, the anti-malarial properties of 400 compounds from the Pathogen Box, provided by Medicines for Malaria Venture (MMV; http://www.mmv.org), were assessed using the analyser. The compounds in this box have activity against pathogens that cause some of the most socioeconomically significant diseases worldwide, including tuberculosis, malaria, sleeping sickness, leishmaniasis, hookworm disease, toxoplasmosis, cryptosporidiosis, and dengue. Of these compounds, 125 have the ability to inhibit the proliferation of *P. falciparum* at the erythrocytic stage, as previously determined using a ‘whole cell’ phenotypic assay [7].

The aim of the current study was to apply the XN-30 analyzer to the discovery and development of anti-malarial drugs. The anti-malarial efficacy of five current anti-malarial drugs (artemisinin, atovaquone, chloroquine, mefloquine, and pyrimethamine) and 400 compounds from the Pathogen Box was characterized using the XN-30 analyzer. The analyser objectively, reproducibly, and easily identified differences in the efficacy of each compound on the parasite. In addition, the compounds were classified into 4 types based on their anti-malarial efficacy. These findings suggest that the XN-30 analyzer is a powerful tool for the characterization of drugs, in addition to being a valuable diagnostic tool that requires no technical experience or expertise.

## Methods

### Parasite strains and culture

*Plasmodium falciparum* 3D7 and W2 were used to assess drug/compound efficacy in vitro. The parasites were cultured in RPMI 1640 medium supplemented with 0.5 g/L l-glutamine, 5.96 g/L HEPES, 2 g/L NaHCO_3_, 50 mg/L hypoxanthine, 10 mg/L gentamicin, 10% heat-inactivated human serum, and RBCs at 3% haematocrit in an atmosphere of 5% CO_2_, 5% O_2_, and 90% N_2_ at 37 °C as described by Trager and Jensen [8]. Ring-form iRBCs were collected using the sorbitol synchronization technique [9]. Briefly, the culture contents were harvested by centrifugation at 840×*g* for 5 min and suspended in a fivefold volume of 5% d-sorbitol (Nacalai Tesque, Kyoto, Japan), and then the cells were washed twice with RPMI 1640 medium to remove d-sorbitol.

### Compounds

Artemisinin (ART), atovaquone (AV), chloroquine diphosphate (CQ), mefloquine hydrochloride (MQ), and pyrimethamine (PYR) were purchased from TCI (Tokyo, Japan). The Pathogen Box compounds were provided by MMV in 96-well plates as 10 mM stock solutions in dimethyl sulfoxide (DMSO) (Nacalai Tesque). MMV also provided the biological activity of these compounds, determined using other screening platforms (ChEMBL-NTD; https://www.ebi.ac.uk/chemblntd), plate layout, and compound details (compound ID, batch ID, trivial name, molecular weight, salt, and cLogP). These data are available online within the Pathogen Box supporting information [10]. ART, AV, MQ, PYR, and the compounds in the Pathogen Box were dissolved in DMSO; CQ was dissolved in water. All compound solutions were stored at − 30 °C and thawed immediately prior to experimentation. Stock solutions were subjected to a maximum of 5 freeze–thaw cycles before being discarded.

### Characterization of parasites treated with anti-malarial drugs

**Fig. 1:**
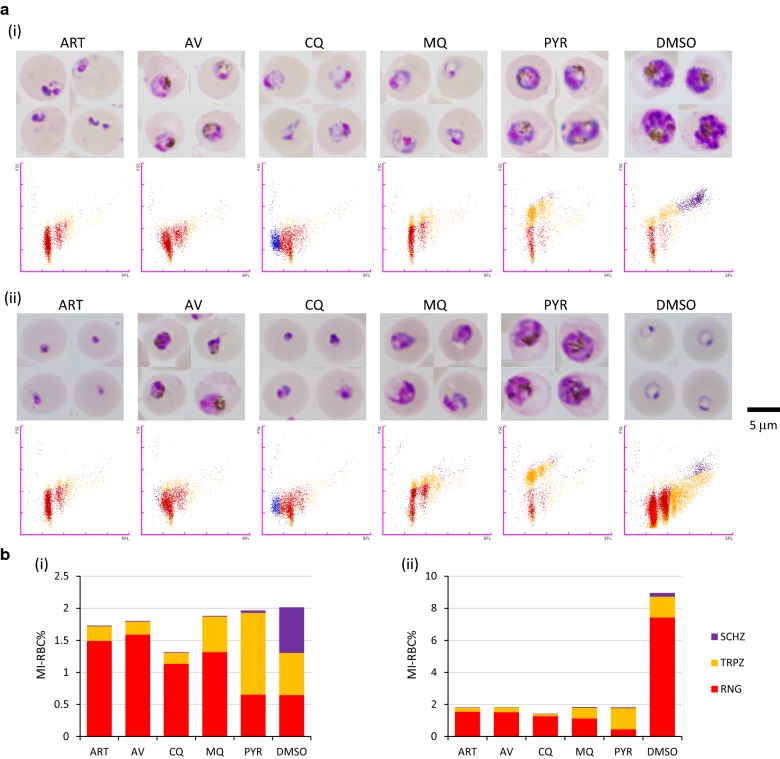
Comparison of parasite outcomes based on observations with microscopy and the XN-30 analyzer. **a** (i) 24 h, (ii) 48 h. Upper and lower panels show microscopic images and M scattergrams obtained from the XN-30 analyzer, respectively. In the microscopic images, the scale bar represents 5 µm. In the scattergrams, the horizontal and vertical axes represent the intensities of side fluorescent light (SFL, which corresponds to DNA content) and forward scattered light (FSC, indicating size of iRBCs), respectively. The colours indicate the following: red, ring-form; orange, trophozoite; purple, schizont; and blue, polychromatic red blood cell (RBC). The colours were assigned based on the default setting of the XN-30 analyzer. **b** Parasitaemia measured with the XN-30 analyzer. Colours are as described above. *ART* artemisinin, *AV* atovaquone, *CQ* chloroquine, *MQ* mefloquine, *PYR* pyrimethamine. The original data obtained from the XN-30 analyzer are provided in Additional file 2: Table S1. All data were acquired using *Plasmodium falciparum* 3D7 with 5 µM of each anti-malarial drug

XN-30 analyzer: The XN-30 analyzer was equipped with the new algorithm for cultured *P. falciparum* parasites (prototype: software version 01-03, build 16) [5] and dedicated reagents were used (CELLPACK DCL, SULFOLYSER, Lysercell M, and Fluorocell M; Sysmex). The culture suspension of 100 µL was diluted with 100 µL phosphate buffered saline and placed in BD Microtainer MAP Microtube for Automated Process K_2_ EDTA 1.0 mg tubes (Becton–Dickinson and Co., Franklin Lakes, NJ, USA). The diluted suspension was then loaded onto the analyser using the auto-sampler option per the manufacturer’s instructions (Sysmex). The XN-30 analyzer recognizes each developmental stage of the parasite using the “M scattergram” and “M (SSC-FSC) scattergram” by analysing side fluorescent light (SFL, corresponds to DNA content), forward scattered light (FSC, indicates the size of iRBCs), and side scattered light (SSC, refers to information about the internal cell structure and its content, e.g. presence of nuclei, granules, etc.) [5, 6]. The feature of representative M scattergrams is explained in Additional file 1: Fig. S1. The M scattergrams showed two populations of ring-forms (e.g. Fig 1a, lower panels); the left population represents RBCs infected by one parasite and the right population represents RBCs infected by two parasites. Once the parasites replicate their DNA, dots are plotted to the right on the M scattergram according to the increase in DNA content [5]. The quantitative parasitaemia of each developmental stage was automatically reported (total, MI-RBC%; ring-form, RNG-RBC%; trophozoite, TRPZ-RBC%; and schizont, SCHZ-RBC%).

Microscopy: A standard thin blood smear was fixed with 100% methanol for 10 min and stained with 10% Giemsa stain (pH 7.2; Merck KGaA, Darmstadt, Germany) for 13 min. The slides were observed at 1000× magnification using the BX50 light microscope (Olympus, Tokyo, Japan).

### Quantification of parasites treated with anti-malarial drugs

Ring-form-synchronized parasites (strains 3D7 and W2, each with approximately 1% parasitaemia) were treated with anti-malarial drugs/compounds for 48 h. The growth inhibition rate was calculated based on the values of SCHZ-RBC% for 24 h and MI-RBC% for 48 h according to the following equation:

$${\text{Growth inhibition }}\left( \% \right)\, = \, 100{-}{{\left( {{\text{test sample}}{-}{\text{positive control}}} \right)} \mathord{\left/ {\vphantom {{\left( {{\text{test sample}}{-}{\text{positive control}}} \right)} {\left( {{\text{negative control}}{-}{\text{positive control}}} \right)}}} \right. \kern-0pt} {\left( {{\text{negative control}}{-}{\text{positive control}}} \right)}}\, \times \, 100.$$

1. For the validation of the assay system, strain 3D7 was treated with 0.5% DMSO for 24 and 48 h. The positive control consisted of 5 µM ART in 0.5% DMSO, and the negative control was saline. Validation indices, coefficient of variation (CV%), signal-to-background ratio (S/B), signal-to-noise ratio (S/N), and Z’-factor were calculated and compared with the minimum pass criteria (CV%) of < 10, S/B of > 2, S/N of > 10, and Z’-factor of > 0.4 [11, 12].
2. For the analysis of the current anti-malarial drugs, strains 3D7 and W2 were treated with the indicated concentration (5.0–0.0015 µM) of the drugs (ART, AV, CQ, MQ, and PYR) in 0.5% DMSO for 48 h. The half-maximal (50%) inhibitory concentration (IC_50_) and 95% confidence interval were determined for each assay by preparing nonlinear regression curves, and a four-parameter logistic curve fit using GraphPad Prism version 5.0 (GraphPad Prism Software, San Diego, CA, USA). The mean and standard deviation (SD) of each drug at the tested concentrations were calculated from the growth inhibition rate.
3. For evaluation of the 400 compounds from the Pathogen Box, strain 3D7 was treated with 5 µM of the respective compound in 0.5% DMSO for 24 and 48 h. The positive and negative controls included 5 µM ART in 0.5% DMSO and 0.5% DMSO alone, respectively. These controls were placed at both edges of a 96-well plate (8 wells each). The growth inhibition rate was calculated as described above.

In the current study, anti-malarial efficacy was considered as > 70% growth inhibition at 24 and/or 48 h. The cut-off for an effective drug was determined by comparison of the analyzer results with the light microscopy observation, namely the efficacy showing < 70% growth inhibition determined by the analyser was difficult to confirm under microscopy. Furthermore, the efficacy was classified into 4 types. A Type I outcome was defined as > 85% of the ratio of RNG-RBC% compared with that of the positive control at 24 h. A Type II outcome was defined as < 90% of the ratio of MI-RBC% compared with that of the negative controls at 24 h. A Type III outcome was defined as > 70% growth inhibition at 24 h, but did not meet the criteria of either a Type I or II outcome (see Fig. 1a, PYR). Finally, a Type IV outcome was defined as > 70% growth inhibition at 48 h but whose growth inhibition rate was < 70% at 24 h.

## Results

### Validation of the assay system

To validate the assay system using the XN-30 analyzer, the effect of 0.5% DMSO on parasite growth was tested. In brief, the ring-form-synchronized parasites were treated with 0.5% DMSO for 48 h, the growth inhibition of parasites treated with 0.5% DMSO was found to be 4.7 ± 3.7% (100.0 ± 0.19% for ART and 0.0 ± 4.2% for saline) at 24 h (Additional file 1: Fig. S2a, b(i)) and 10.9 ± 3.3% (100.0 ± 0.50% for ART and 0.0 ± 1.8% for saline) at 48 h (Additional file 1: Fig. S2c, d(i)). These findings suggest that the effect of 0.5% DMSO is acceptable for this assay system. The validation indices, CV%, S/B, S/N, and Z′-factor values were 0.78, 21.2, 25.8, and 0.88 at 24 h (Additional file 1: Fig. S2a, b(ii)) and 0.30, 9.2, 27.2, and 0.87 at 48 h (Additional file 1: Fig. S2c and d(ii)), respectively. These values were within the minimum criteria, suggesting that the XN-30 analyzer is competent for drug screening.

### Microscopy and the XN-30 analyzer-based quantification of parasites treated with anti-malarial drugs

Microscopy showed that the majority of control parasites treated with DMSO progressed to trophozoite and schizont at 24 h and to new ring-form at 48 h (Fig. 1a, DMSO). These observations correlated well with the XN-30 analyzer measurements (Fig. 1a, b, DMSO). In contrast, at 24 h, microscopy showed parasites arrested at various stages of growth: shrunken morphology (ART, CQ, and MQ), early trophozoite (AV) and mid-trophozoite (PYR). Notably, both CQ- and MQ-treated parasites showed no haemozoin formation (Fig. 1a(i)). The M scattergrams also showed that the ATR-, AV-, and MQ-treated parasites were arrested at the ring-form (red dots), CQ exhibited both ring-form (red dots) and polychromatic RBCs (blue dots), and PYR treatment halted the development of parasites at the trophozoite (orange dots) (Fig. 1a(i) lower panels). At 48 h, the ART- and CQ-treated parasites were shrunken with no further development, whereas AV-, MQ-, and PYR-treated parasites reached early to mid-trophozoite with abnormal morphology (Fig. 1a(ii), upper panels). The M scattergrams of drug-treated parasites showed no further development compared with those at 24 h. These results suggested that these drugs arrested parasite growth or killed parasites. Increased polychromatic RBCs (blue dots) and/or dispersed dots apparent in the CQ-treated parasites may be caused by competition between CQ and the DNA staining dye in Fluorocell M (see Discussion). No increase in DNA content was noted and thus, the drug-treated parasites did not undergo DNA replication. As the XN-30 analyzer simultaneously displays parasitaemia and the occurrence of DNA replication, these data provide further clues as to which developmental stage is the most affected by the drug (Fig. 1b and Additional file 2: Table S1).

### Measurement of drug efficacy using the XN-30 analyzer

**Fig. 2:**
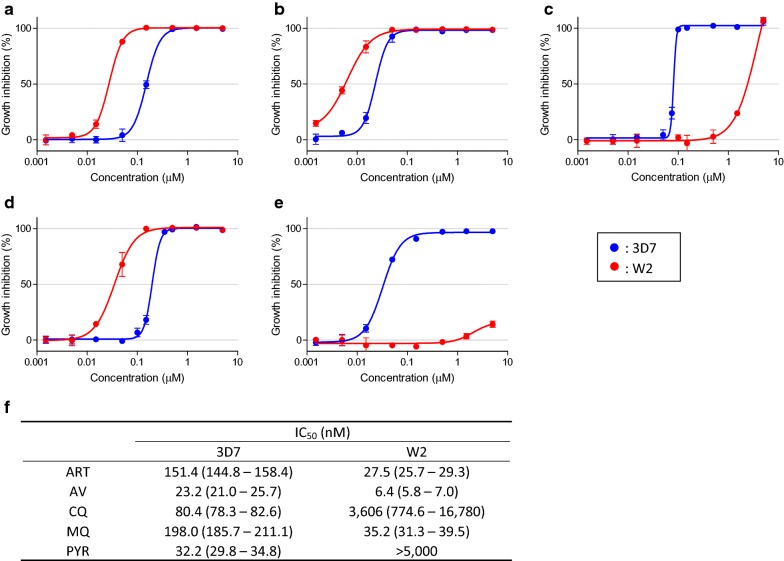
Measurement of efficacy of anti-malarial drugs on *P. falciparum* parasites. The growth inhibition rate of *P. falciparum* 3D7 (blue) and W2 (red) treated with anti-malarial drugs. **a** ART, **b** AV, **c** CQ, **d** MQ, **e** PYR, **f** the summary of IC_50_ values. The values in parentheses give 95% confidence intervals. Mean, standard deviation and IC_50_ were calculated based on the data of three independent wells. The original data obtained from the XN-30 analyzer are provided in Additional file 2: Table S2

The ability of the XN-30 analyzer to measure drug efficacy on parasites was assessed using the strains 3D7 and W2. Respective strains and drugs indicated strongly fitted sigmoidal curves (Fig. 2a–e), and the median SD at all tested concentrations was 1.6 (minimum 0.15, maximum 8.7), suggesting that the variation in values measured with the XN-30 analyzer was acceptably low for the comparison of drug efficacy (Fig. 2a–e and Additional file 2: Table S2). The IC_50_ for strains 3D7 and W2 was 151.4 nM and 27.5 nM for ART, 23.2 nM and 6.4 nM for AV, 80.4 nM and 3606 nM for CQ, 198.0 nM and 35.2 nM for MQ, and 32.2 nM and > 5000 nM for PYR (Fig. 2f), respectively. The high resistance of strain W2 against CQ and PYR was consistent with a previous report that suggested the resistance was caused by three point mutations in the dihydrofolate reductase gene [13].

### Characterization of parasites treated with compounds from the Pathogen Box

**Fig. 3:**
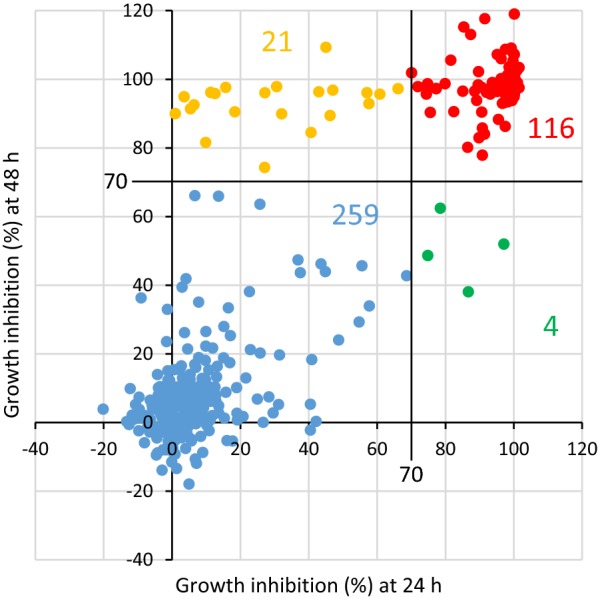
Efficacy of compounds in the Pathogen Box on *P. falciparum* 3D7. The scatter-plot of growth inhibition rates of parasites treated with 5 µM of each of the 400 compounds from the Pathogen Box. The horizontal and vertical axes indicate the growth inhibition rate of parasites treated with the compounds for 24 and 48 h, respectively. Growth inhibition of 70% was defined as a cut-off point for compound efficacy. Colour indications are as follows: red, > 70% (24 h) and > 70% (48 h); blue, < 70% (24 h) and < 70% (48 h); orange, < 70% (24 h) and > 70% (48 h); and green, > 70% (24 h) and < 70% (48 h)

The parasitaemia of parasites treated with 5 µM of each of the 400 compounds included in the Pathogen Box was measured using the XN-30 analyzer. Growth inhibition of greater than 70% at 24 and/or 48 h was defined as *P. falciparum*-inhibiting activity. Of the 400 compounds assayed, 141 compounds were effective, based on the aforementioned cut-off of 70% growth inhibition. Specifically, 116 were effective at both 24 and 48 h; 4 were effective at 24 h, but not at 48 h; and 21 were effective at 48 h, but not at 24 h. As such, 120 and 137 were effective at 24 and 48 h, respectively (Fig. 3 and Table 1).

**Table 1:** List of 141 compounds exhibiting anti-malarial activity against *P. falciparum* 3D7

Growth inhibition (%)							
Plate	#	Position	Compound ID	24 h	48 h	Type	Disease set
A	#1	A02	MMV010764	66.1	97.3	IV	MAL
A	#2	B03	MMV000907	97.6	108.7	III	MAL
A	#3	C02	MMV084603	98.6	103.6	III	MAL
A	#4	C04	MMV688888	100.1	100.1	III	TUB
A	#5	D04	MMV661713	100.1	107.2	II	TUB
A	#6	E03	MMV676350	99.8	107.6	II	MAL
A	#7	F02	MMV026020	91.5	117.6	III	MAL
A	#8	H08	MMV676474	86.7	38.1	III	TUB
B	#1	A03	MMV652003	97.3	93.2	I	KIN
B	#2	A04	MMV000062	100.1	99.0	I	REF
B	#3	A05	MMV006372	100.1	100.1	III	MAL
B	#4	A09	MMV020623^b^	99.9	96.8	I	MAL
B	#5	A10	MMV020512	95.7	97.7	I	MAL
B	#6	B03	MMV676604	57.0	96.1	IV	KIN
B	#7	B07	MMV020982	100.1	97.9	II	MAL
B	#8	B08	MMV020120	100.1	97.9	I	MAL
B	#9	B10	MMV007638	89.5	98.4	III	MAL
B	#10	B11	MMV021057	100.1	97.2	I	MAL
B	#11	C07	MMV020136^b^	100.1	98.1	I	MAL
B	#12	C08	MMV020710^b^	99.9	97.9	I	MAL
B	#13	C09	MMV020517	82.4	90.5	III	MAL
B	#14	C10	MMV019721	100.1	98.1	I	MAL
B	#15	D02	MMV020537	99.9	100.6	I	MAL
B	#16	D05	MMV000063	89.8	83.0	III	REF
B	#17	D08	MMV020520^b^	100.1	98.4	I	MAL
B	#18	D09	MMV019234	100.1	95.6	II	MAL
B	#19	D10	MMV016136	99.9	96.9	I	MAL
B	#20	E07	MMV676442	100.1	98.8	I	MAL
B	#21	E09	MMV024397	100.1	100.6	I	MAL
B	#22	E10	MMV019807	99.9	98.5	I	MAL
B	#23	F02	MMV019189	5.4	91.4	IV	MAL
B	#24	F05	MMV637229	98.8	97.6	I	TRI
B	#25	F07	MMV020321	78.5	62.4	III	MAL
B	#26	F08	MMV019087	95.5	88.3	III	MAL
B	#27	F09	MMV676528	99.6	98.1	I	MAL
B	#28	F10	MMV020320	40.6	84.5	IV	MAL
B	#29	F11	MMV085210^b^	100.1	96.6	I	MAL
B	#30	G05	MMV689480	74.8	48.7	III	REF
B	#31	G07	MMV006239^b^	100.1	98.7	I	MAL
B	#32	G08	MMV000858^b^	99.9	96.9	I	MAL
B	#33	G09	MMV006741	100.1	103.7	II	MAL
B	#34	H02	MMV676602	77.3	97.3	III	KIN
B	#35	H03	MMV000016^c^	99.9	100.4	I	REF
B	#36	H05	MMV668727	15.7	97.6	IV	ONC
B	#37	H08	MMV006901	100.1	119.0	II	MAL
B	#38	H09	MMV020391^b^	99.9	97.2	I	MAL
C	#1	A05	MMV688122	90.8	77.9	III	TUB
C	#2	B02	MMV020388	100.4	96.7	II	MAL
C	#3	B03	MMV688547	90.8	85.8	III	KIN
C	#4	B05	MMV687749	99.6	96.7	II	TUB
C	#5	C03	MMV688283	81.6	105.5	II	KIN
C	#6	C09	MMV688361	97.5	86.3	III	KIN
C	#7	C11	MMV022236	89.1	93.8	III	MAL
C	#8	D05	MMV687248	100.0	100.8	II	TUB
C	#9	D11	MMV1030799	99.6	99.0	III	MAL
C	#10	E10	MMV021375	96.6	93.0	II	MAL
C	#11	F03	MMV688179	27.1	74.3	IV	KIN
C	#12	F04	MMV023969	100.4	98.5	II	TUB
C	#13	F08	MMV687807	71.9	97.8	III	TUB
C	#14	G03	MMV675993	100.0	98.0	I	CRY
C	#15	G04	MMV021660	100.4	101.9	II	TUB
C	#16	G06	MMV687273	97.1	52.0	III	TUB
C	#17	G11	MMV688703	97.5	95.6	II	TOX
C	#18	H07	MMV024311	100.0	97.6	I	TUB
D	#1	A02	MMV026468	74.4	95.7	III	MAL
D	#2	A03	MMV020670	93.7	99.1	III	MAL
D	#3	A04	MMV023953	30.6	97.8	II	MAL
D	#4	A05	MMV010576	99.7	99.9	I	MAL
D	#5	A06	MMV032967	97.7	98.3	II	MAL
D	#6	A07	MMV031011	89.7	102.2	II	MAL
D	#7	A09	MMV688362	99.7	98.5	III	KIN
D	#8	A11	MMV026356	100.3	98.4	III	MAL
D	#9	B02	MMV011511	95.4	98.2	II	MAL
D	#10	B04	MMV007471	99.5	93.8	I	MAL
D	#11	B05	MMV024829	99.7	105.4	II	MAL
D	#12	B07	MMV022029	95.1	107.2	II	MAL
D	#13	B09	MMV688180	27.2	96.1	IV	KIN
D	#14	B10	MMV024035	77.3	97.3	II	MAL
D	#15	C03	MMV006833	93.4	97.8	III	MAL
D	#16	C04	MMV026490	74.4	97.6	III	MAL
D	#17	C05	MMV687246	85.3	115.2	II	MAL
D	#18	C07	MMV024114	79.9	98.7	II	MAL
D	#19	D03	MMV020081^b^	100.0	99.3	I	MAL
D	#20	D04	MMV026550	6.4	92.5	IV	MAL
D	#21	D07	MMV023860	85.1	96.5	III	MAL
D	#22	D09	MMV023949	93.1	95.7	II	MAL
D	#23	D11	MMV024406	87.4	113.1	II	MAL
D	#24	E02	MMV023233	100.9	102.6	II	MAL
D	#25	E03	MMV085230	86.5	80.2	III	MAL
D	#26	E04	MMV085071^a^	99.2	98.8	III	MAL
D	#27	E05	MMV659004	32.0	89.9	IV	KIN
D	#28	E06	MMV676260	90.5	97.6	II	MAL
D	#29	E08	MMV032995	75.5	90.3	III	MAL
D	#30	E09	MMV688279	100.0	95.6	I	KIN
D	#31	E10	MMV688271	100.3	97.6	III	KIN
D	#32	F03	MMV011765	3.5	94.9	IV	MAL
D	#33	F04	MMV024937	92.0	96.2	II	MAL
D	#34	F05	MMV085499	99.2	98.9	I	MAL
D	#35	F06	MMV023985	70.1	101.9	IV	MAL
D	#36	F07	MMV024195	45.0	109.3	II	MAL
D	#37	F11	MMV687812	96.3	106.0	II	TUB
D	#38	G02	MMV007803	100.0	94.9	III	MAL
D	#39	G03	MMV001059^b^	100.3	100.2	I	MAL
D	#40	G04	MMV011691	94.9	96.9	III	MAL
D	#41	G05	MMV676877	99.2	100.5	III	MAL
D	#42	G06	MMV663250	99.7	101.0	III	MAL
D	#43	G11	MMV676411	88.5	96.5	III	TUB
D	#44	H02	MMV007133	11.3	96.2	IV	MAL
D	#45	H03	MMV022478	100.9	98.6	II	MAL
D	#46	H05	MMV676881	0.9	90.0	IV	MAL
D	#47	H06	MMV024443	47.0	96.8	IV	MAL
D	#48	H08	MMV023388	95.1	97.6	II	MAL
D	#49	H09	MMV675968	96.3	100.2	II	CRY
D	#50	H11	MMV688980^b^	100.0	97.2	I	MAL
E	#1	A02	MMV011229	101.0	99.0	I	MAL
E	#2	A06	MMV688775	90.6	90.5	III	REF
E	#3	A08	MMV393144	98.3	93.5	III	MAL
E	#4	A11	MMV019993	99.3	99.6	I	MAL
E	#5	B02	MMV687794	9.8	81.6	IV	MAL
E	#6	B08	MMV023183	99.2	109.1	II	MAL
E	#7	C04	MMV671636	100.0	99.2	I	ONC
E	#8	C08	MMV687765	99.7	99.7	II	TUB
E	#9	C09	MMV020165	91.4	84.0	III	MAL
E	#10	D02	MMV688766	74.8	98.7	III	SCH
E	#11	D04	MMV667494	100.4	99.6	I	MAL
E	#12	D05	MMV028694	42.9	96.3	IV	MAL
E	#13	D07	MMV688345	57.6	92.9	IV	TOX
E	#14	D08	MMV010545	98.1	101.2	III	MAL
E	#15	E02	MMV020289	100.1	99.0	III	MAL
E	#16	E04	MMV634140	100.4	97.1	I	MAL
E	#17	E05	MMV030734	12.6	95.8	IV	MAL
E	#18	E09	MMV407834	18.4	90.5	IV	MAL
E	#19	F02	MMV019551	99.1	96.3	I	MAL
E	#20	F09	MMV026313	46.2	89.4	IV	MAL
E	#21	G04	MMV021013	99.3	100.0	III	TUB
E	#22	G05	MMV392832	101.5	103.4	II	MAL
E	#23	G06	MMV688754	100.2	98.9	III	KIN
E	#24	G08	MMV658988	60.8	95.7	IV	KIN
E	#25	G09	MMV084864	101.5	97.5	II	MAL
E	#26	H05	MMV688978	100.0	102.4	I	REF
E	#27	H10	MMV688550	95.1	96.4	III	KIN

*MAL* malaria, *TUB* tuberculosis, *KIN* kinetoplastids, *REF* reference compounds, *TRI* trichuriasis, *ONC* onchocerciasis, *CRY* cryptosporidiosis, *TOX* toxoplasmosis, *SCH* schistosomiasis

^a^Positive compounds described in Tong et al. [19]

^b^Positive compounds described in Dennis et al. [20]

^c^Mefloquine

**Fig. 4:**
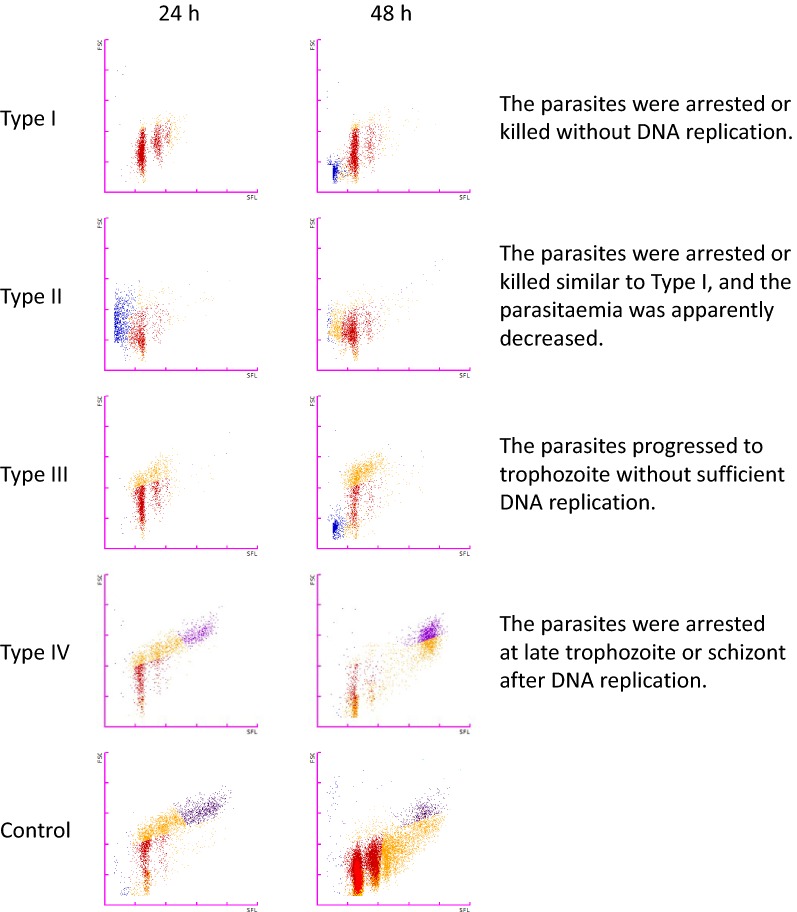
Classification of the effective compounds analysed with the XN-30 analyzer. Typical M scattergrams of Type I to IV outcomes: Type I, MMV676442; Type II, MMV661713; Type III, MMV688762; and Type IV, MMV026550. In the scattergrams, the horizontal and vertical axes represent the intensities of SFL, which corresponds to the DNA content, and FSC, which indicates iRBC size, respectively. The colours indicate the following: red, ring-form; orange, trophozoite; purple, schizont; and blue, polychromatic RBC. The colours were assigned based on the default setting of the XN-30 analyzer

The efficacy of the 141 effective compounds was classified into four major types based on the parasitaemia/scattergram data obtained from the analyzer. Type I indicated that the parasites were arrested or killed without DNA replication. Microscopy showed that the parasites treated with the compounds were shrunken, similar to that of ART-treated parasites (see Fig. 1a, ART). Type II indicated that the parasites were arrested or killed, similar to Type I and parasitaemia was apparently decreased (see Fig. 1a, CQ). Type III indicated that the parasites progressed to trophozoite without sufficient DNA replication (see Fig. 1a, PYR). Finally, Type IV indicated that the parasites were arrested at late trophozoite or schizont after DNA replication (see Additional file 1: Fig. S6). In summary, 38 compounds showed Type I effects (27.0%), 37 showed Type II effects (26.2%), 45 showed Type III effects (31.9%), and 21 showed Type IV effects (14.9%). No DNA replication was observed in Types I-III in approximately 85.0% of the 141 effective compounds (Fig. 4 and Table 1).

## Discussion

The XN-30 analyzer was primarily developed to detect parasites in clinical blood samples [4, 5]. Previous studies have demonstrated that the analyser is capable of analysing parasites in in vitro culture [5] and rodent malaria parasites in vivo [6]. The present study demonstrated that the analyser can be further applied to evaluate anti-malarial drugs. The analyser was able to evaluate and characterize the efficacy of current anti-malarial drugs (Fig. 1). In addition, although Fig. 1a presents four typical microscopic images of drug treatment, several morphological variations can be observed even in the controls treated with DMSO (Additional file 1: Fig. S4). Comparison of the data from the XN-30 analyzer with that of microscopy suggested that the analyser was able to more objectively represent drug efficacy. The conventional techniques that evaluate parasite growth, such as measurement of uptake of ^3^H-hypoxanthine, colourimetric measurement of lactate dehydrogenase activity, and various flow cytometry techniques as well as microscopy with Giemsa-staining [2], are time-consuming and require technical experience or expertise, suggesting that the XN-30 analyzer easily differentiates anti-malarial efficacy.

The IC_50_ of all the current anti-malarial drugs in strains 3D7 and W2 was relatively higher than that of other studies (e.g., ART, 10.78 ± 2.01 nM; CQ, 12.63 ± 2.34 nM; MQ, 12.29 ± 2.01; and PYR, 10.01 ± 2.13 for strain 3D7 in [14]; Fig. 2). In addition, strain 3D7 (CQ- and PYR-sensitive) was more tolerant to ART, AV, and MQ than that of strain W2. However, outcomes similar to those of the XN-30 analyzer were demonstrated by the microscopic observation. This suggests that these differences are due to culture conditions (e.g., incubation period and medium components) but not the assay system.

In this study, the XN-30 analyzer revealed the ART-, AV-, and MQ-treated parasites were in the ring-form at 24 h and even at 48 h (Fig. 1a, ART, AV, and MQ). In addition, parasitaemia (SCHZ-RBC% at 24 h and MI-RBC% at 48 h) apparently showed that the growth of parasites was arrested at ring-form because trophozoite (orange/TRPZ-RBC%) and schizont (purple/SCHZ-RBC%) did not appear at 24 h and total parasitaemia (MI-RBC%) did not increase at 48 h (Fig. 1, ART, AV, and MQ and Additional file 2: Table S1). These facts indicate that the analyser can define the effect of these drugs on the parasites before DNA replication. In addition, Type II outcomes observed in CQ-treated parasites appeared as polychromatic RBCs (blue dots) on the M scattergram (Fig. 1a, CQ). This observation does not indicate increment of polychromatic RBC and/or decrement of the DNA content, but suggest the two following possibilities. First, binding competition may occur between the test compound and the DNA staining dye in Fluorocell M. Second, fragmentation and efflux of genomic DNA may occur with the test compound (Additional file 1: Fig. S5). Preliminary biochemical studies showed that CQ was able to inhibit DNA and RNA syntheses, but its intercalation with DNA did not explain the anti-malarial activity or the selective toxicity [15–18]. These facts suggest that the Type II outcome was due to the intercalation of CQ in the parasite DNA, which competes with the DNA staining dye. Of the 141 effective compounds, 37 presented the Type II outcome (Table 1 and Additional file 1: Fig. S3b). Although it is unclear whether these compounds similarly exclude DNA staining dye or caused DNA fragmentation (Additional file 1: Fig. S5), the XN-30 analyzer will provide new insights into the mechanism of action of the compounds. In contrast, impedance of haemozoin formation by CQ and MQ was observed by microscopy but not using the analyser (Fig. 1a), indicating that the analyzer was unable to detect this phenomenon. The Type III outcome indicated that the parasites progressed to trophozoite without sufficient DNA replication.

This phenomenon could be generated from two possibilities: the compound completely arrested the growth of parasite at trophozoite and delayed growth progression. The latter would be demonstrated by observating a parasite treated with the compound at different concentrations. The Type IV outcome implied that parasite development was arrested at the late trophozoite or schizont after DNA replication. This arrest of parasites was similarly observed by microscopy at 48 h after compound treatment (Additional file 1: Fig. S6).

To further confirm the performance of the XN-30 analyzer in this assay system, the data obtained in this study were compared with reported outcomes. A previous study indicated that the compound MMV085071 disrupts *P. falciparum*’s digestive vacuole and yielded anti-malarial efficacy [19]. The current study also demonstrated that this compound inhibited the growth of parasites by 99.2%, and it was within Type III classification (Table 1 and Additional file 1: Fig. S3c). Another study demonstrated that 11 compounds (MMV000858, MMV001059, MMV006239, MMV020081, MMV020136, MMV020391, MMV020520, MMV020623, MMV020710, MMV085210, and MMV688980) perturbed the putative parasite Na^+^ efflux P-type ATPase, PfATP4 [20]. In the present study, all eleven compounds exhibited 99% growth inhibition, and it was within Type I classification (Table 1 and Additional file 1: Fig. S3a). In comparison, a non-effective compound (MMV676269) showed only 0.9% growth inhibition in this study (Table 1). In addition, the reference compound (MMV000016, or mefloquine) presented outcomes similar to that of MQ (Additional file 1: Fig. S3a; compare with Fig. 1a, MQ). These results indicate that the XN-30 analyzer performs reliably in drug screening.

Finally, of the 125 anti-plasmodial compounds assigned in the Pathogen Box [7], 97 exhibited more than 70% growth inhibition and 28 exhibited less than 70% inhibition in this study (Table 1). Of these 28 compounds, 16 had previously been described as efficacious at less than 2 µM of IC_50_ for strain 3D7 but did not show growth inhibition activity at 5 µM [7]. As these compounds also exerted low growth inhibition efficacy in parasites as assessed by microscopy, this discrepancy is not due to the performance of the XN-30 analyzer but was due to the different assay conditions.

The XN-30 analyzer requires at least 70 µL of test sample [5]. This amount is suitable for assays using 96-well plates, but not for the measurement of smaller amounts of sample, encountered using 384-well and 1536-well plates. Therefore, the analyzer should be improved in the future for larger sample sets.

## Conclusion

This study demonstrated that the XN-30 analyzer objectively, reproducibly, and easily evaluated and characterized the efficacy of anti-malarial compounds. Furthermore, the efficacy was classified into 4 types. These findings suggested that the XN-30 analyzer is a powerful tool for drug discovery and development, as well as diagnostic usage.

## Additional files

**Additional file 1: Fig. S1.** Representative M scattergram of *in vitro* cultured sample. The horizontal and vertical axes indicate intensities of side fluorescent light (SFL, which corresponds to DNA content) and forward scattered light (FSC, indicating size of iRBCs), respectively. The colours indicate the following: red, ring-form; orange, trophozoite; purple, schizont; and blue, polychromatic RBC. The colours were assigned based on the default setting of the XN-30 analyzer. The scattergram was cited from Fig. 1a(i), DMSO). **Fig. S2.** Validation in the assay system. (**a** and **b**) 24 h, (**c** and **d**) 48 h. (**a** and **c**) The scatter-plot of the growth inhibition rate. The growth inhibition rate was calculated based on SCHZ-RBC% at 24 h and MI-RBC% at 48 h (see also Methods). The colours indicate the following: blue, 0.5% DMSO; dark red, positive control (5 µM artemisinin); and dark blue, negative control (saline). (**b**(i) and **d**(i)) The growth inhibition rate. (**b**(ii) and **d**(ii)) The values of validation indices. Abbreviations are as follows: CV %, coefficient of variation; S/B, signal-to-background ratio; and S/N, signal-to-noise ratio. **Fig. S3.** M scattergrams of the effective compounds, related to Figs. 3 and 4 and Table 1. (**a**) Type I, (**b**) Type II, (**c**) Type III, (**d**) Type IV. The left and right panels indicate scattergrams at 24 and 48 h, respectively. *, **, and, ** represent effective compounds described in Tong et al. [19] and Dennis et al. [20], and the reference compound mefloquine, respectively. The colours indicate the following: red, ring-form; orange, trophozoite; purple, schizont; and blue, polychromatic RBC. The colours were assigned based on the default setting of the XN-30 analyzer; however, these may be misclassified after compound treatment as described in the Discussion. **Fig. S4.** Microscopic images of parasites treated with anti-malarial drugs, related to Fig. 1. (**a**) ART, (**b**) CQ, (**c**) DMSO. Sixteen representative images were randomly selected. Scale bar represents 5 µm. **Fig. S5.** Two possibilities for the generation of a Type II outcome. Possibility 1: The test compound competed with the DNA staining dye in Fluorocell M, as insufficiently stained DNA are likely to show low DNA content. Possibility 2: The test compound fragmented genomic DNA and the DNA was flowed out; the efflux of fragmented DNA reduced the DNA content. **Fig. S6.** Microscopic images of Type IV outcome, related to Figs. 3 and 4 and Table 1. Typical microscopic images of parasites treated with MMV026550, MMV011765, MMV024443, or MMV030734 after 48 h of incubation. Scale bar represents 5 µm.

**Additional file 2: Table S1.** Parasitaemia of each parasite culture treated with the anti-malarial drugs. **Table S2.** Growth inhibition rate of parasites treated with the anti-malarial drugs.

## Abbreviations

RBC: red blood cell
iRBC: infected RBC
FSC: forward scattered light
SFL: side fluorescent light
SSC: side scattered light
ART: artemisinin
AV: atovaquone
CQ: chloroquine
MQ: mefloquine
PYR: pyrimethamine
DMSO: dimethyl sulfoxide
SD: standard deviation
CV: coefficient of variation
S/B: signal-to-background ratio
S/N: signal-to-noise ratio
MMV: Medicines for Malaria Venture
RIMD: Research Institute for Microbial Diseases
