# Supplementary material for: Application of the automated haematology analyzer XN-30 for discovery and development of anti-malarial drugs
Source: Malar J. 2019 Jan 14;18:8. doi: 10.1186/s12936-019-2642-0 (PMC6332852; doi:10.1186/s12936-019-2642-0)

Fig. S1

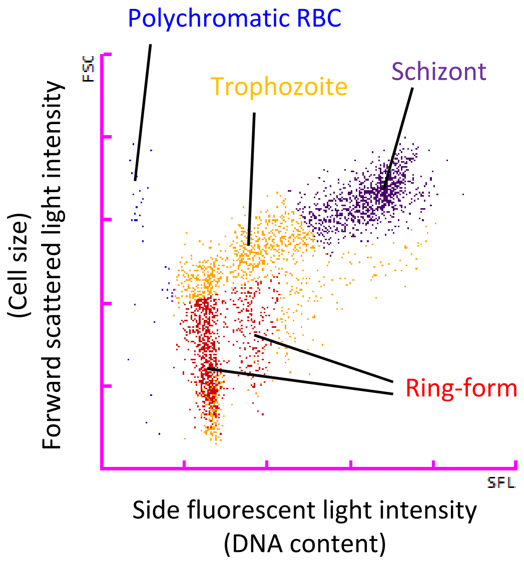

Polychromatic RBC
Trophozoite
Schizont
Ring-form
FSC
SFL
(Cell size)
Forward scattered light intensity
Side fluorescent light intensity
(DNA content)

Fig. S2

a

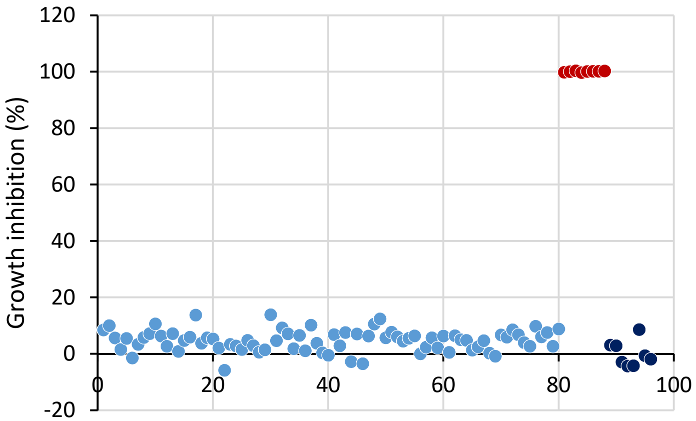

b

(i)

	Growth inhibition (%)
ART	100.0±0.19%
Saline	0.0±4.2%
DMSO	4.7±3.7%

(ii)

	Result	Validation index
CV %	0.78	<20
S/B ratio	21.2	>3
S/N ratio	25.8	<10
Z'-factor	0.88	>0.4

c

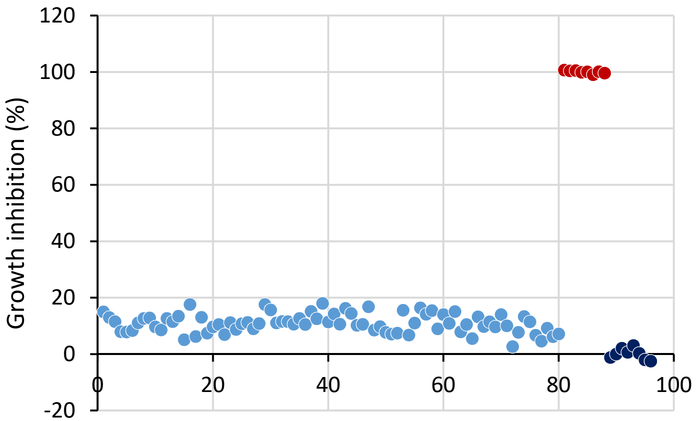

d

(i)

	Growth inhibition (%)
ART	100.0±0.50%
Saline	0.0±1.8%
DMSO	10.9±3.3%

(ii)

	Result	Validation index
CV %	0.30	<20
S/B ratio	9.2	>3
S/N ratio	27.2	<10
Z'-factor	0.87	>0.4

Fig. S3a

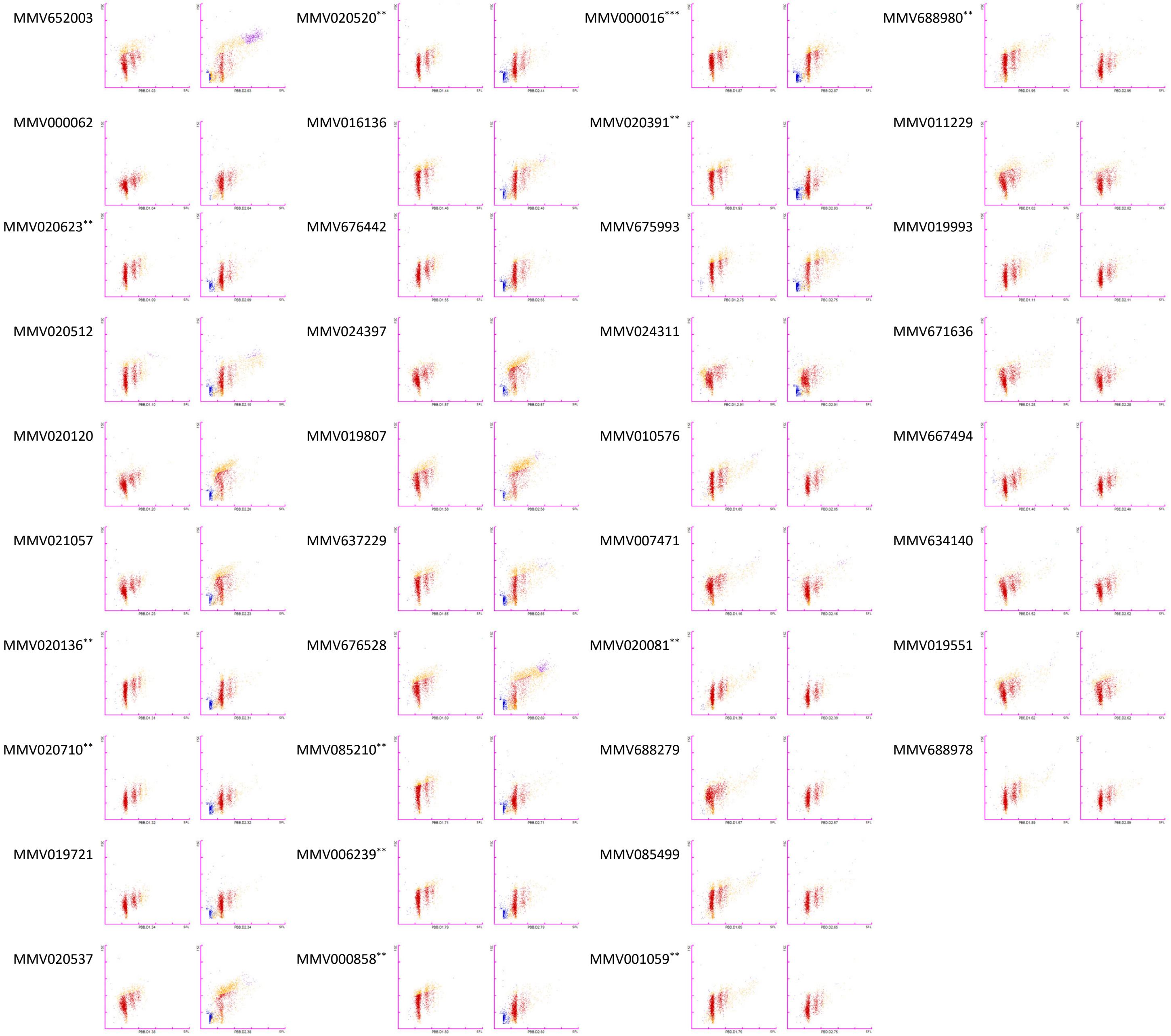

MMV652003
MMV020520**
MMV000016***
MMV688980**
MMV000062
MMV016136
MMV020391**
MMV011229
MMV020623**
MMV676442
MMV675993
MMV019993
MMV020512
MMV024397
MMV024311
MMV671636
MMV020120
MMV019807
MMV010576
MMV667494
MMV021057
MMV637229
MMV007471
MMV634140
MMV020136**
MMV676528
MMV020081**
MMV019551
MMV020710**
MMV085210**
MMV688279
MMV688978
MMV019721
MMV006239**
MMV085499
MMV020537
MMV000858**
MMV001059**

Fig. S3b (continued)

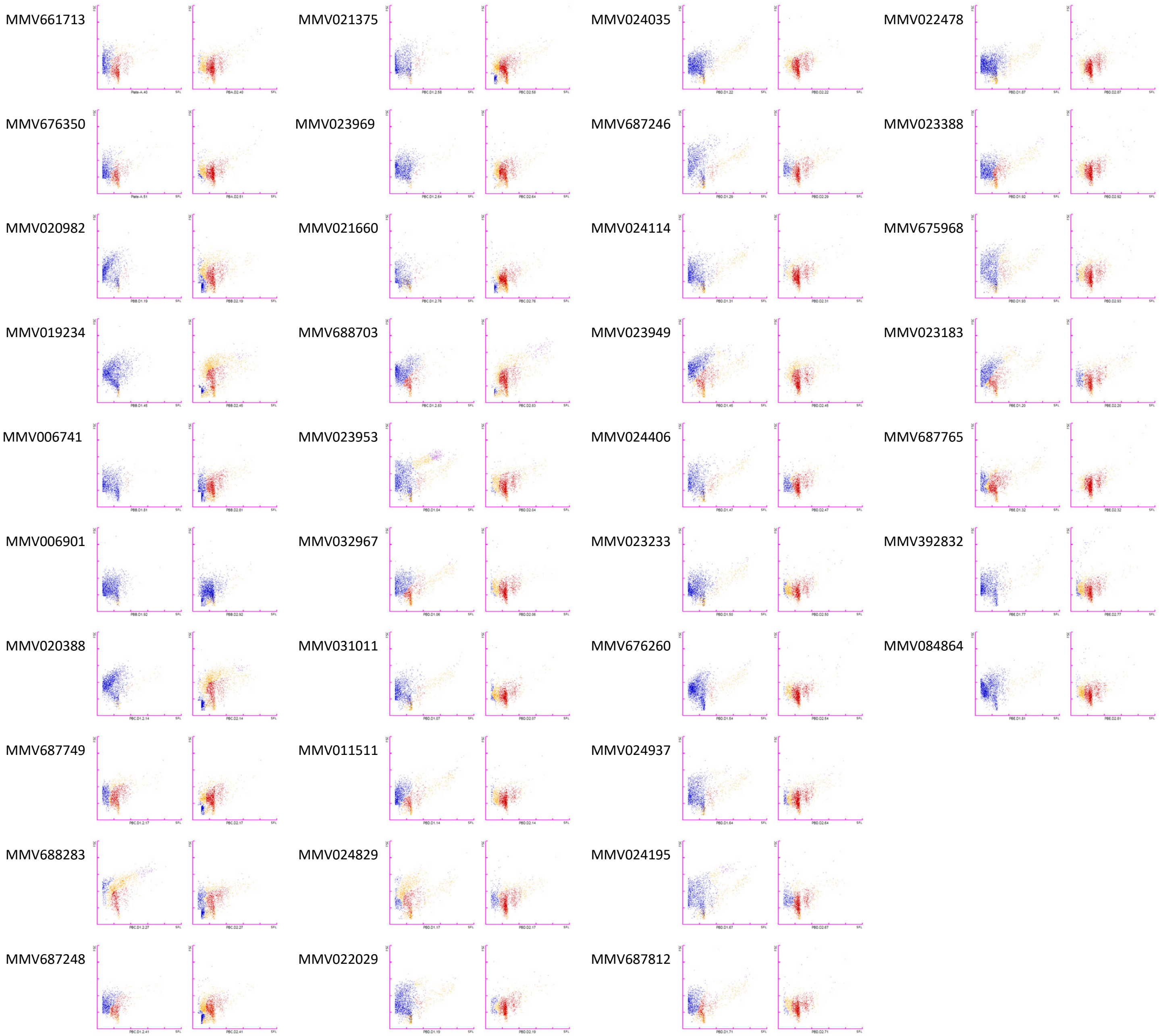

Fig. S3c (continued)

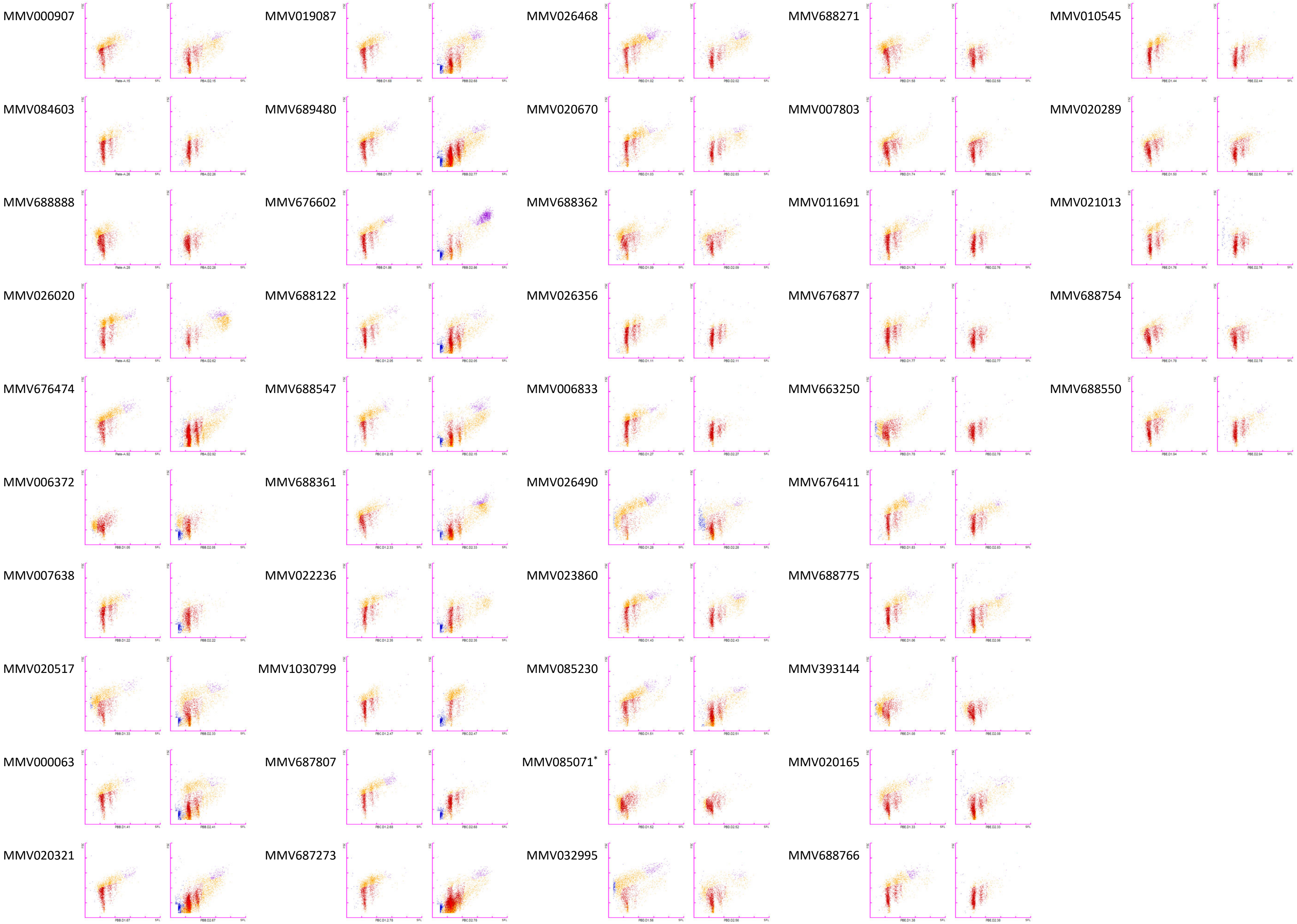

Fig. S3d (continued)

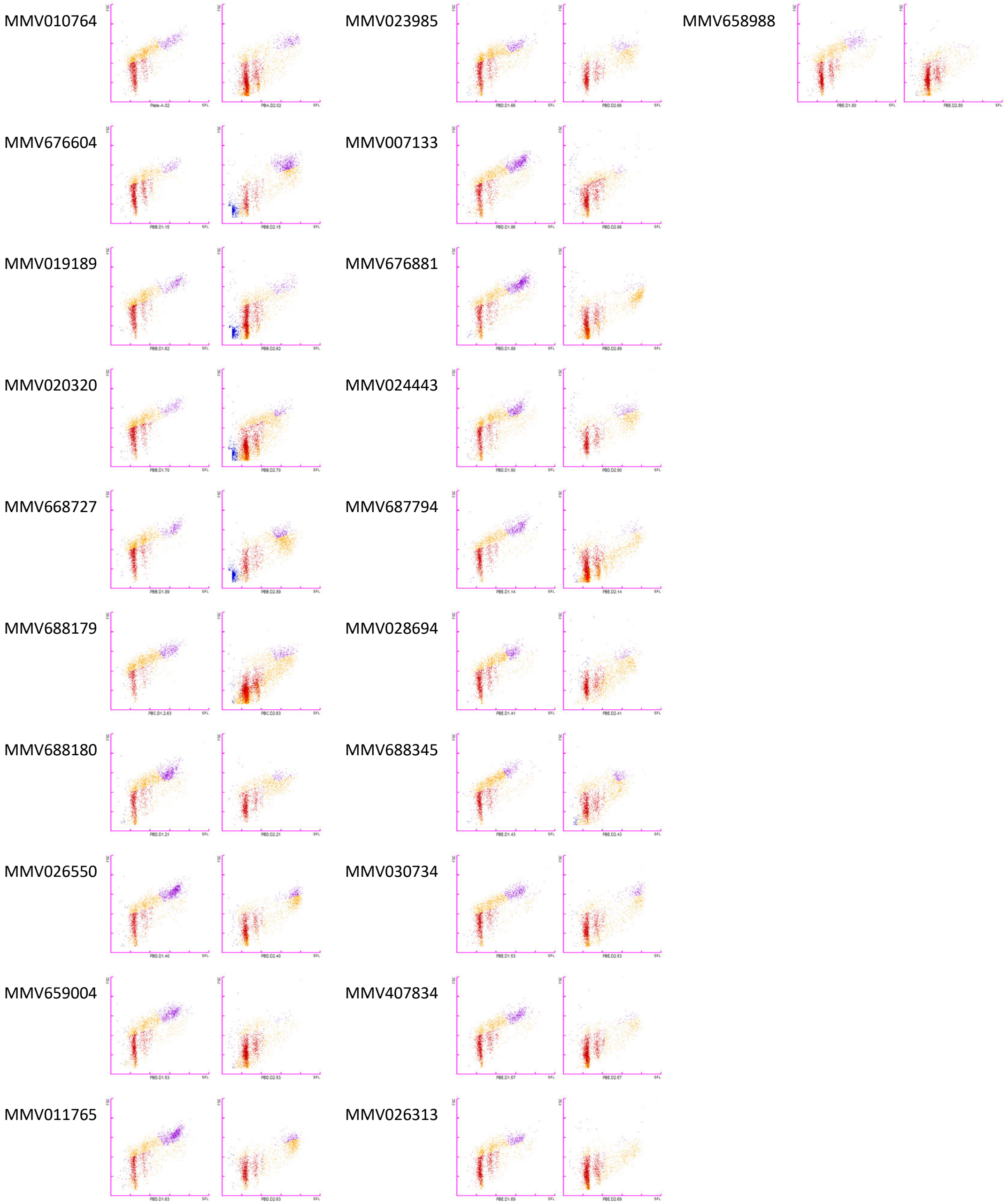

Fig. S4

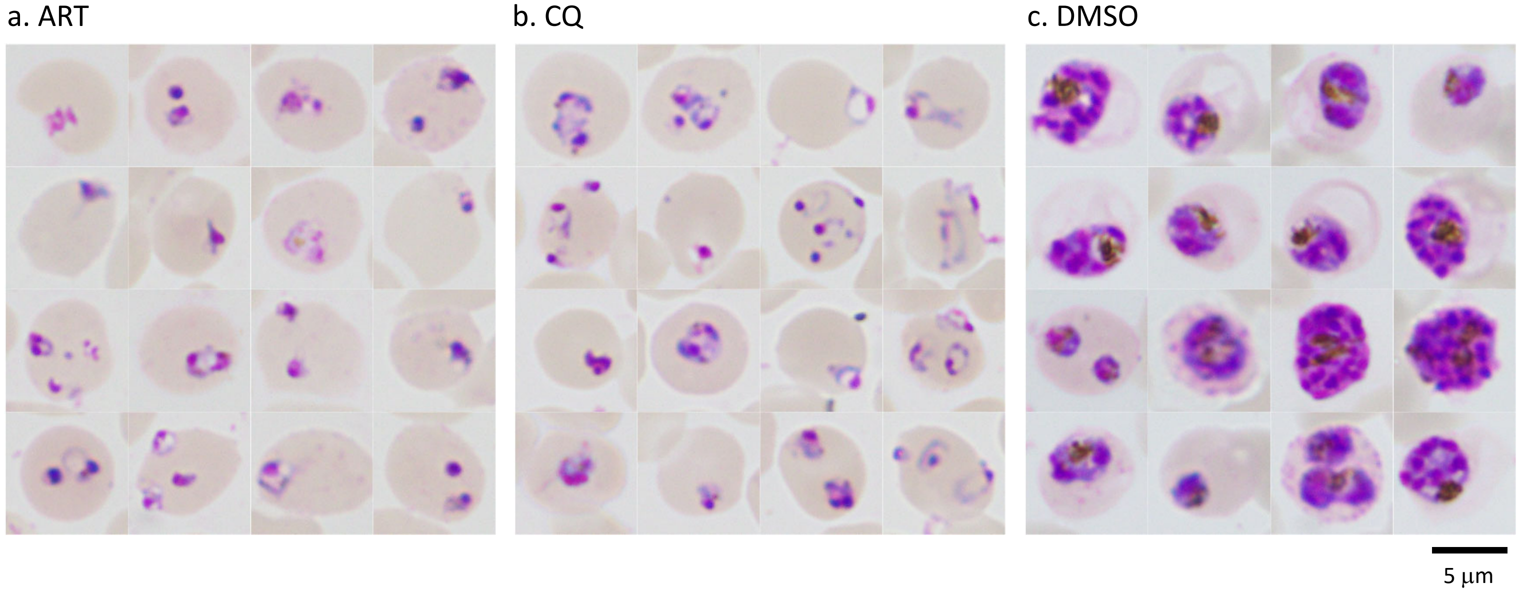

Fig. S5

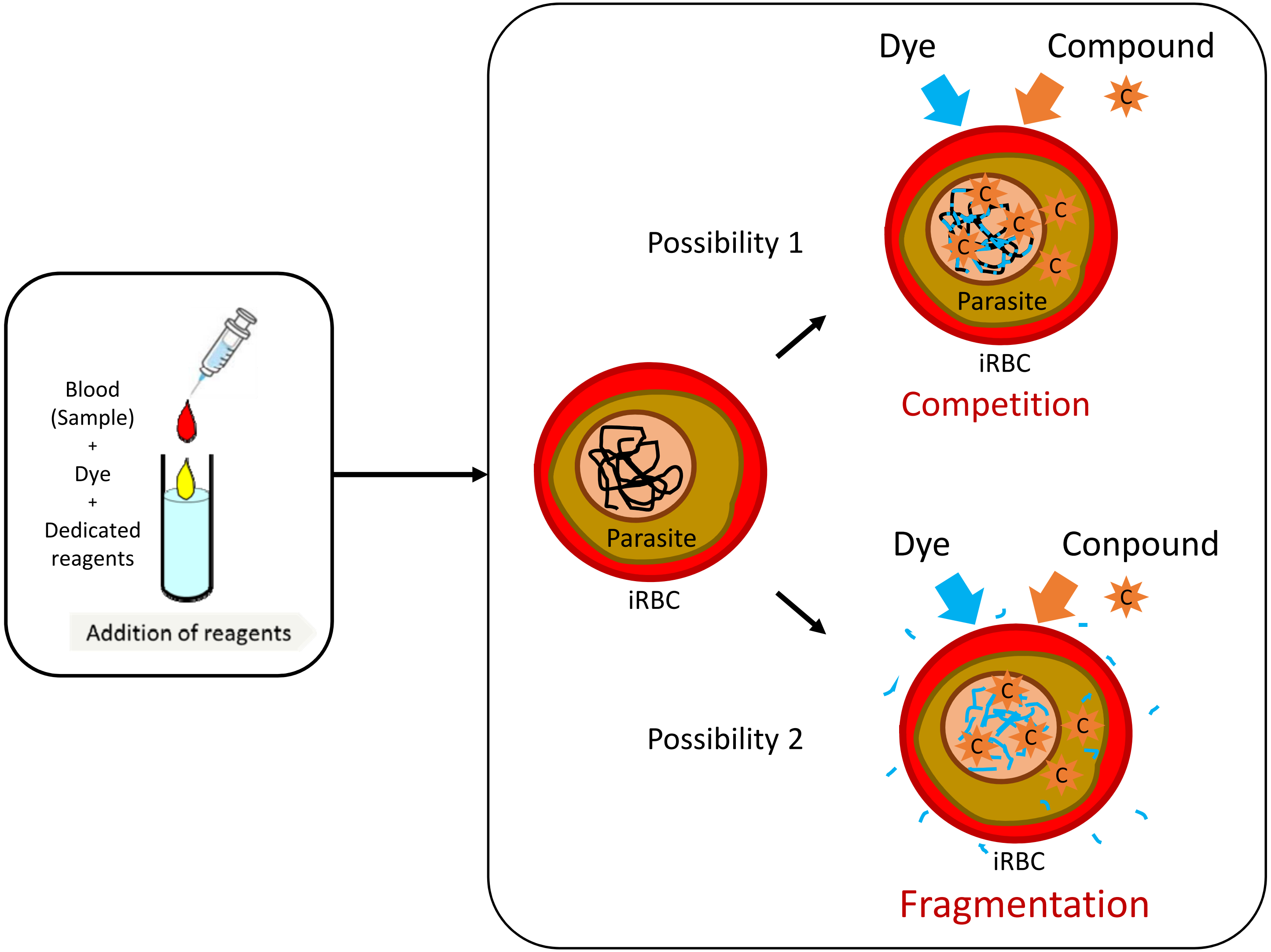

Blood
(Sample)
+
Dye
+
Dedicated
reagents
Addition of reagents
Parasite
iRBC
Possibility 1
Dye
Compound
C
Parasite
iRBC
Competition
Possibility 2
Dye
Conpound
C
iRBC
Fragmentation

Fig. S6

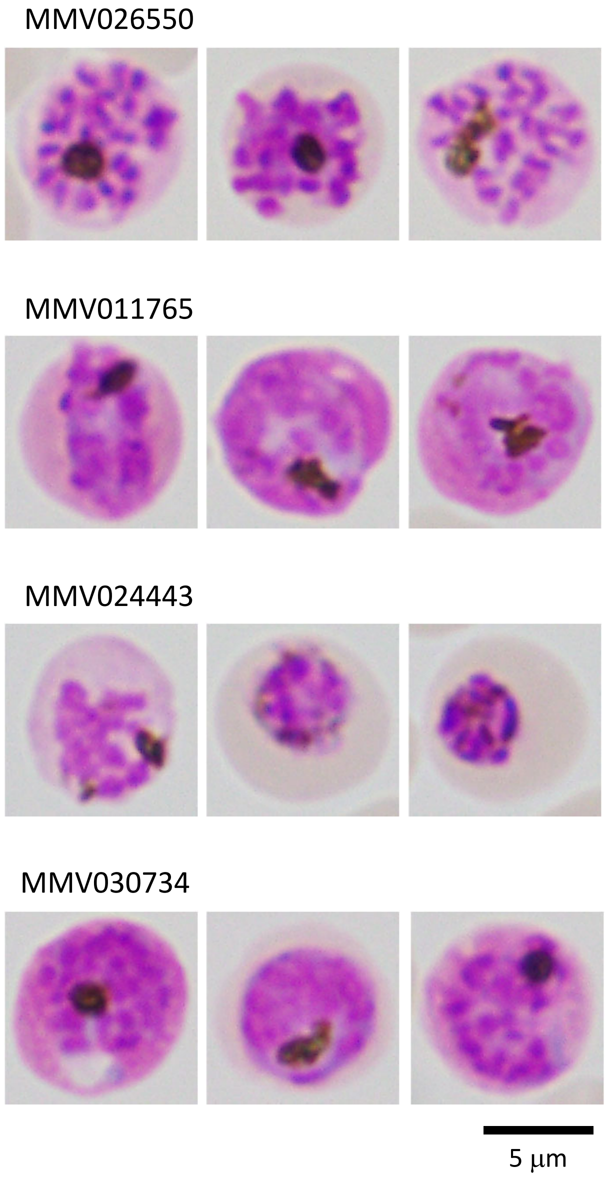

Supplement: Supplementary file 1 — Additional file 1: Fig. S1. Representative M scattergram of in vitro cultured sample. The horizontal and vertical axes indicate intensities of side fluorescent light (SFL, which corresponds to DNA content) and forward scattered light (FSC, indicating size of iRBCs), respectively. The colours indicate the following: red, ring-form; orange, trophozoite; purple, schizont; and blue, polychromatic RBC. The colours were assigned based on the default setting of the XN-30 analyzer. The scattergram was cited from Fig. 1a(i), DMSO). Fig. S2. Validation in the assay system. (a and b) 24 h, (c and d) 48 h. (a and c) The scatter-plot of the growth inhibition rate. The growth inhibition rate was calculated based on SCHZ-RBC% at 24 h and MI-RBC% at 48 h (see also Methods). The colours indicate the following: blue, 0.5% DMSO; dark red, positive control (5 µM artemisinin); and dark blue, negative control (saline). (b(i) and d(i)) The growth inhibition rate. (b(ii) and d(ii)) The values of validation indices. Abbreviations are as follows: CV %, coefficient of variation; S/B, signal-to-background ratio; and S/N, signal-to-noise ratio. Fig. S3. M scattergrams of the effective compounds, related to Figs. 3 and 4 and Table 1. (a) Type I, (b) Type II, (c) Type III, (d) Type IV. The left and right panels indicate scattergrams at 24 and 48 h, respectively. *, **, and, ** represent effective compounds described in Tong et al. [19] and Dennis et al. [20], and the reference compound mefloquine, respectively. The colours indicate the following: red, ring-form; orange, trophozoite; purple, schizont; and blue, polychromatic RBC. The colours were assigned based on the default setting of the XN-30 analyzer; however, these may be misclassified after compound treatment as described in the Discussion. Fig. S4. Microscopic images of parasites treated with anti-malarial drugs, related to Fig. 1. (a) ART, (b) CQ, (c) DMSO. Sixteen representative images were randomly selected. Scale bar represents 5 µm. F [file 12936_2019_2642_MOESM1_ESM.pdf]
