# Supplementary material for: Application of the automated haematology analyzer XN-30 for discovery and development of anti-malarial drugs
Source: Malar J. 2019 Jan 14;18:8. doi: 10.1186/s12936-019-2642-0 (PMC6332852; doi:10.1186/s12936-019-2642-0)
Supplement: Supplementary file 2 — Additional file 2: Table S1. Parasitaemia of each parasite culture treated with the anti-malarial drugs. Table S2. Growth inhibition rate of parasites treated with the anti-malarial drugs. [file 12936_2019_2642_MOESM2_ESM.pdf]

Table S1: Parasitaemia of each parasite culture treated with anti-malarial drugs

	RBC%	ART	AV	CQ	MQ	PYR	DMSO
24 h	RNG	1.493	1.588	1.134	1.317	0.652	0.647
	TRPZ	0.228	0.205	0.172	0.554	1.277	0.656
	SCHZ	0.010	0.010	0.010	0.012	0.038	0.710
48 h	RNG	1.546	1.520	1.270	1.128	0.452	7.427
	TRPZ	0.261	0.295	0.142	0.663	1.321	1.296
	SCHZ	0.016	0.013	0.011	0.050	0.045	0.234

ART, artemisinin; AV, atovaquone; CQ, chloroquine; MQ, mefloquine; and PYR, pyrimethamine.
RNG, ring-form; TRPZ, trophozoite; and SCHZ, schizont.

Table S2: Growth inhibition rate of parasites treated with anti-malarial drugs

3D7 strain

ART*

Concentration	Growth inhibition (%)	Mean	SD**
	98.8		
5 μM	99.0	99.5	0.90
	100.8		
	100.1		
1.5 μM	101.0	100.2	0.64
	99.5		
	98.5		
0.5 μM	100.4	99.3	0.80
	99.1		
	52.6		
0.15 μM	45.9	49.4	2.8
	49.8		
	0.62		
0.05 μM	1.2	4.1	4.5
	10.5		
	-2.5		
0.015 μM	3.4	0.13	2.5
	-0.49		
	5.0		
0.005 μM	-0.32	0.94	3.0
	-1.9		
	-1.0		
0.0015 μM	-0.25	-0.64	0.31
	-0.67		

AV*

Concentration	Growth inhibition (%)	Mean	SD**
	97.9		
5 μM	98.7	98.5	0.40
	98.8		
	98.3		
1.5 μM	99.2	98.5	0.57
	97.8		
	96.4		
0.5 μM	97.0	97.2	0.84
	98.4		
	98.5		
0.15 μM	99.9	99.0	0.65
	98.6		
	86.8		
0.05 μM	96.2	92.7	4.2
	95.0		
	22.4		
0.015 μM	22.7	19.6	4.2
	13.7		
	7.9		
0.005 μM	5.7	6.2	1.3
	4.9		
	-4.5		
0.0015 μM	4.4	0.53	3.7
	1.7		

CQ*

Concentration	Growth inhibition (%)	Mean	SD**
	108.3		
5 μM	108.0	106.2	2.8
	102.3		
	99.9		
1.5 μM	100.8	101.1	1.2
	102.7		
	101.6		
0.5 μM	103.6	102.3	0.94
	101.6		
	100.9		
0.15 μM	100.1	100.3	0.40
	100.0		
	99.1		
0.1 μM	99.1	99.0	0.19
	98.7		
	29.4		
0.075 μM	19.1	23.8	4.2
	23.0		
	-0.9		
0.005 μM	6.4	4.3	3.7
	7.5		
	0.3		
0.0015 μM	1.1	2.2	2.1
	5.2		
	-2.1		
0.005 μM	-2.2	0.3	3.5
	5.2		
	-1.4		
0.0015 μM	-1.0	-0.7	0.7
	0.3		

MQ*

Concentration	Growth inhibition (%)	Mean	SD**
	99.7		
5 μM	98.7	98.7	0.85
	97.6		
	100.8		
1.5 μM	101.6	101.6	0.59
	102.3		
	97.4		
0.5 μM	101.3	99.3	1.6
	99.0		
	96.7		
0.35 μM	97.8	97.0	0.54
	96.6		
	16.9		
0.15 μM	14.9	18.1	3.2
	22.5		
	7.0		
0.1 μM	10.5	6.8	3.1
	2.8		
	-1.6		
0.05 μM	-1.3	-0.87	0.9
	0.3		
	0.0		
0.015 μM	1.8	0.70	0.8
	0.3		
	3.8		
0.005 μM	1.8	0.5	3.3
	-4.0		
	0.4		
0.0015 μM	3.4	0.3	2.6
	-2.9		

PYR*

Concentration	Growth inhibition (%)	Mean	SD**
	98.2		
5 μM	97.4	97.8	0.31
	97.7		
	98.3		
1.5 μM	97.4	97.7	0.45
	97.4		
	97.1		
0.5 μM	97.7	97.3	0.28
	97.0		
	89.2		
0.15 μM	90.2	90.8	1.7
	93.2		
	73.8		
0.05 μM	71.3	72.4	1.0
	72.2		
	14.4		
0.015 μM	8.2	10.6	2.7
	9.1		
	-4.5		
0.005 μM	5.1	-0.19	4.0
	-1.1		
	-2.5		
0.0015 μM	-4.4	-2.0	2.1
	0.8		

W2 strain

ART*

Concentration	Growth inhibition (%)	Mean	SD**
	99.9		
5 μM	101.4	100.2	0.94
	99.2		
	100.2		
1.5 μM	100.4	100.4	0.15
	100.6		
	100.1		
0.5 μM	100.3	100.5	0.42
	101.0		
	100.4		
0.15 μM	100.9	100.6	0.23
	100.5		
	89.0		
0.05 μM	87.5	88.1	0.69
	87.7		
	17.5		
0.015 μM	10.0	14.0	3.0
	14.4		
	3.9		
0.005 μM	2.6	4.2	1.5
	6.2		
	-5.1		
0.0015 μM	3.4	-0.074	3.6
	1.5		

AV*

Concentration	Growth inhibition (%)	Mean	SD**
	98.7		
5 μM	98.0	98.7	0.55
	99.4		
	99.8		
1.5 μM	100.0	99.2	0.93
	97.9		
	100.3		
0.5 μM	97.8	99.2	1.0
	99.6		
	99.1		
0.15 μM	97.8	98.5	0.54
	98.6		
	98.2		
0.05 μM	99.3	98.7	0.45
	98.6		
	88.3		
0.015 μM	77.6	83.3	4.4
	84.1		
	47.7		
0.005 μM	41.6	44.3	2.5
	43.7		
	12.1		
0.0015 μM	17.3	14.7	2.1
	14.8		

CQ*

Concentration	Growth inhibition (%)	Mean	SD**
	109.3		
5 μM	104.4	107.6	2.3
	109.2		
	25.3		
1.5 μM	23.7	23.7	1.3
	22.2		
	7.0		
0.5 μM	5.3	2.7	4.9
	-4.2		
	4.4		
0.15 μM	-9.9	-3.2	5.9
	-4.0		
	4.7		
0.05 μM	0.9	1.8	2.1
	-0.1		
	3.1		
0.015 μM	1.7	-1.0	4.8
	-7.7		
	1.9		
0.005 μM	-3.7	-1.1	2.3
	-1.6		
	0.1		
0.0015 μM	0.6	-1.3	2.3
	-4.6		

MQ*

Concentration	Growth inhibition (%)	Mean	SD**
	97.9		
5 μM	98.6	98.9	0.89
	100.1		
	101.0		
1.5 μM	100.2	100.8	0.40
	101.1		
	101.0		
0.5 μM	100.6	100.8	0.17
	100.7		
	99.6		
0.15 μM	99.8	100.0	0.42
	100.6		
	78.7		
0.05 μM	57.3	67.9	8.7
	67.5		
	14.0		
0.015 μM	14.8	14.4	0.35
	14.6		
	5.0		
0.005 μM	-2.6	-0.36	3.8
	-3.5		
	3.2		
0.0015 μM	-1.5	0.32	2.1
	-0.7		

PYR*

Concentration	Growth inhibition (%)	Mean	SD**
	14.8		
5 μM	16.7	14.3	2.3
	11.3		
	2.7		
1.5 μM	6.2	3.5	2.0
	1.6		
	-0.5		
0.5 μM	-0.9	-1.6	1.3
	-3.4		
	-4.2		
0.15 μM	-6.0	-5.7	1.2
	-7.0		
	-4.2		
0.05 μM	-4.4	-4.7	0.60
	-5.5		
	2.7		
0.015 μM	-6.3	-4.7	5.5
	-10.5		
	2.3		
0.005 μM	3.7	0.13	4.1
	-5.6		
	2.4		
0.0015 μM	0.3	0.33	1.7
	-1.7		

*: ART, artemisinin; AV, atovaquone; CQ, chloroquine; MQ, mefloquine; and PYR, pyrimethamine

**: SD, standard deviation
